# Predictors of Length-of-Stay Among Transcatheter Aortic Valve Replacement Patients Using a Supervised Machine Learning Algorithm

**DOI:** 10.1016/j.jacadv.2025.101902

**Published:** 2025-06-24

**Authors:** Gregory L. Judson, Jeff Luck, Skye Lawrence, Rakan Khaki, Harsh Agrawal, Krishan Soni, Kirsten Tolstrup, Vijayadithyan Jaganathan, Vaikom S. Mahadevan

**Affiliations:** a Department of Medicine Division of Cardiology, University of California-San Francisco, San Francisco, California, USA; b Department of Medicine Division of Cardiology, Maine Medical Center, Portland, Maine, USA; c Biome Analytics, Chicago, Illinois, USA; d University of Massachusetts Chan Medical School, Worcester, Massachusetts, USA

**Keywords:** length of stay, machine learning, TAVR

## Abstract

**Background:**

Length of stay following transcatheter aortic valve replacement (TAVR) continues to improve, but significant gaps remain in predicting the length of stay following TAVR.

**Objectives:**

This study aimed to develop a novel machine learning (ML) algorithm that would facilitate the understanding of the predictors of early and late hospital discharge in patients who have undergone TAVR.

**Methods:**

Using the Biome data set, 9,172 outpatient TAVR procedures were analyzed from 21 centers between 2017 and 2021 across the United States. Supervised random forest ML algorithms were developed to identify variables involved in short length of stay (SLOS) (length of stay <36 hours) and long length of stay (LLOS) (length of stay ≥72 hours) in a 70% sample of the Biome data set. The models were then tested on the remaining 30% of the data set and results compared to standard multivariable models in predicting LOS.

**Results:**

Twenty and 22 variables were identified and included as important predictors for the SLOS and LLOS multivariable models, respectively. The predictive power of both the SLOS (sensitivity 0.81, specificity 0.70, area under the curve [AUC] 0.82) and LLOS (sensitivity 0.45, specificity 0.94, AUC 0.85) ML models were more robust than the standard multivariable model (SLOS AUC 0.65, LLOS AUC 0.65). Our study uncovered several previously unreported predictors for length of stay following TAVR, such as procedural duration, postprocedure physical therapy, and procedure day of the week.

**Conclusions:**

ML algorithms may have an important role in identifying novel predictors of short and prolonged length of stay following TAVR. These efforts may facilitate targeted quality improvement programs to decrease length of stay post-TAVR.

Transcatheter aortic valve replacement (TAVR) is now the most common form of aortic valve replacement procedure in the United States and is approved for low-, intermediate-, and high-risk patients with severe aortic stenosis.^1^ Since its approval by the Food and Drug Administration (FDA) in 2011, complication rates, length of stay (LOS), and overall mortality in TAVR have consistently improved.^2^ Given the increasing volume of TAVR procedures performed in the United States, there has been intense focus on minimizing hospital LOS post-TAVR. The need to limit LOS has only been exacerbated by the COVID-19 pandemic where difficult decisions often must be made in regard to resource utilization in the face of a global pandemic.^3^

In order to better understand how specific changes in patient characteristics or the provision of TAVR contributed to shorter LOS and early vs late hospital discharge, we developed a novel machine learning (ML) algorithm within the large multicenter, retrospective observational Biome data set. Test characteristics of this algorithm were then internally validated and compared to a standard multivariable model and the relative importance and direction of each predictor for short length of stay (SLOS) (<36 hours) and long length of stay (LLOS) (≥72 hours) determined.

## Methods

### Study population

The study cohort was assembled from the Biome (Chicago, IL) multicenter data repository. Briefly, the Biome data set contains aggregated clinical and financial data across 21 U.S. hospital sites that perform TAVR. Thirteen hospitals in the data set identify as teaching hospitals. Site-specific TAVR volume varied across participating hospitals with 12 centers performing 100+ TAVRs per year, 6 performing 50 to 99 TAVRs per year, and 3 sites performing 1 to 50 TAVRs per year. Fourteen hospitals in the data set were located in the Western US, 4 in the Midwest, and 3 in the Northeast. Clinical data fields are populated via linkage with the datasheets for the National Cardiovascular Data Registry Transcatheter Valve Therapy registry. Additional data fields are populated from item cost accounting data provided by Biome member hospitals. The study received proper ethical oversight and was approved by the local research ethics committee.

Outpatient TAVR cases from January 2017 through December 2021 were identified by limiting cases to those with <24 hours admission time preprocedure. Cases were excluded if preprocedural LOS could not be determined, or if missing or incomplete constituted >5% of the total data, or if the patient died during the procedural admission. A total of 12,184 TAVR procedures were identified with 3,012 cases excluded yielding 9,172 cases for the analysis.

Variables of interest were determined a priori as potentially relevant from prior literature review or potential clinical relevance.

### Statistical design

SLOS was defined as LOS <36 hours, with LOS determined from preprocedure admission time to discharge time. LLOS was defined as ≥72 hours. The median value was imputed for missing values of Kansas City Cardiomyopathy Questionnaire (KCCQ-12) score (10%) and contrast volume (11%). Missing values of the 5-meter walk test (27%) were imputed to either the median or the maximum value (60 seconds), with probability based on the proportion of observed cases having the maximum value.

A 70% random sample of the cohort was used for model development; the remaining 30% was reserved as the test data set. Random forest models were used because they perform well with large numbers of continuous and categorical predictors, can identify nonlinear relationships between predictors and outcomes, and are robust to overfitting and outliers.^4^^,^^5^ They also facilitate feature identification, that is, identifying the contribution of predictors to observed outcomes.

Random forest models were developed separately for SLOS and LLOS using the training data set. The LLOS model was chained, with the SLOS model prediction included as a predictor.

Recursive feature elimination with cross-validation (RFECV) graphs were produced to identify the ideal number of predictors for each model. Backward elimination was performed to retain the most significant predictors, and the relative importance of each predictor was quantified using Gini scores. Sensitivity, specificity, and area under the curve (AUC) measurements were determined using the test data set. SHapley Additive exPlanations values, which are a way to explain the output of a ML model, were calculated and plotted for significant predictors. Finally, random forest model results were compared against multivariate logistic regression results for SLOS and LLOS, with each logistic regression model containing the same predictors as the corresponding random forest model to which it was compared. RFECV graphs were produced to identify the optimal number of predictors for each model (20 for SLOS and 21 plus SLOS prediction for LLOS). Backward elimination was performed to retain the optimal number of most significant predictors, and the relative importance of each predictor was quantified using Gini scores (Central Illustration).

**Central Illustration:**
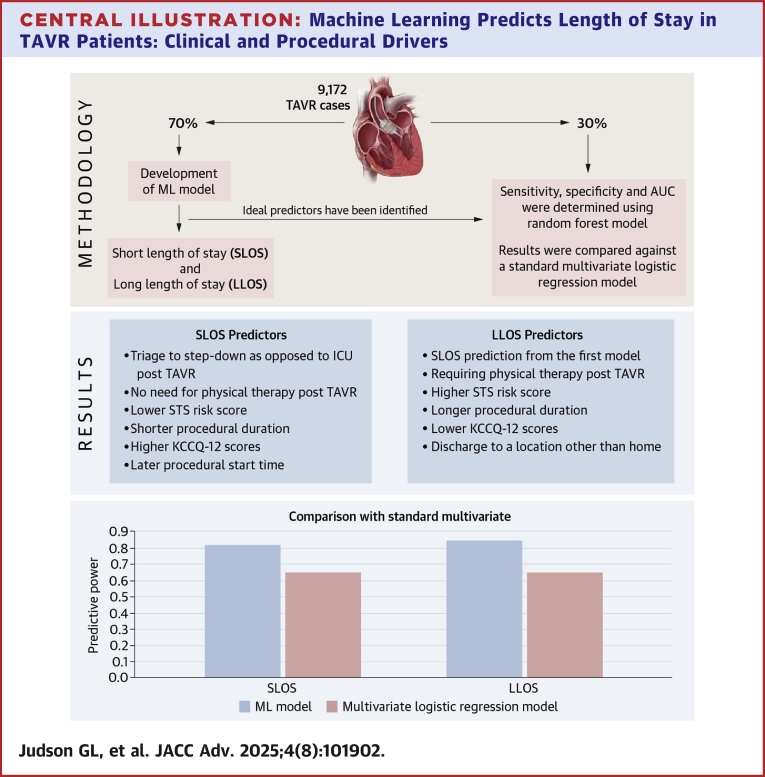
**Machine Learning Predicts Length of Stay in TAVR Patients: Clinical and Procedural Drivers** Using data from 9,172 TAVR procedures across 21 U.S. centers (2017-2021), ML models were developed to predict short length of stay (SLOS <36 hours) and long length of stay (LLOS ≥72 hours). The models identified novel predictors, including post-TAVR physical therapy, procedural duration, and discharge disposition. SLOS was associated with factors such as triage to step-down units, lower STS risk scores, and higher KCCQ-12 scores, while LLOS was linked to physical therapy needs and higher STS scores. The random forest models outperformed traditional multivariate models in predictive accuracy, suggesting ML can enhance discharge planning and quality improvement efforts in TAVR care ML = machine learning; other abbreviations as in Figure 3.

## Results

### Study population

**Figure 1:**
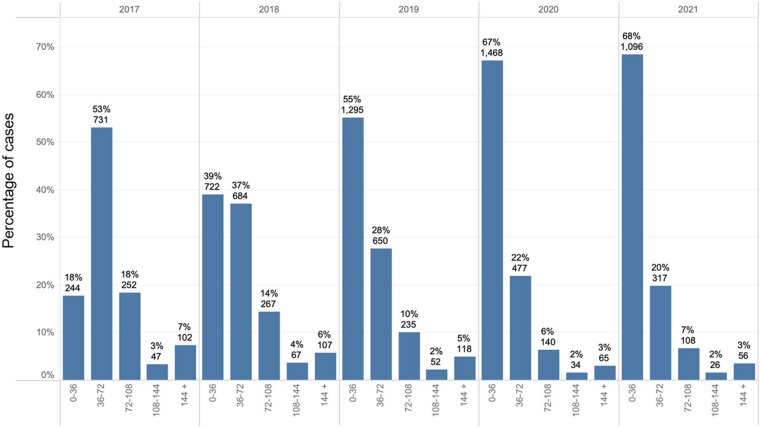
**Percentage of Cases Based on Length of Stay in the BIOME Database From 2017 to 2021** Distribution of cases based on hospital length of stay (LOS) from 2017 to 2021 in the BIOME database. Patients were categorized by LOS duration to assess trends in clinical resource utilization and patient outcomes over time.

A total of 9,172 patients were included in our analysis, with 4,719 having SLOS and 1,651 identified as LLOS. The study cohort had an average age of 79.4 years old, was 43% female, had an average Society of Thoracic Surgeons (STS) score of 4.3%, and TAVR was performed in 97% of cases via transfemoral access. Patient characteristics as well as procedural data are further summarized in Table 1. P values for continuous variables are based on *t*-tests, and for categorical variables on chi-square tests. Figure 1 outlines the distribution of LOS from 2017 to 2021. Across the study period, overall TAVR volume increased year over year with the percentage of SLOS increased (18% of cases in 2017 to 68% of cases in 2021) with a corresponding decrease in LLOS (28% in 2017, 12% in 2021).

**Table 1:** Description of Study Cohort

Continuous Predictors	Overall Cohort (n = 9,172) Mean (SD)	Met SLOS <36 (n = 4,719) Mean (SD)	Did Not Meet SLOS ≥36 (n = 4,453) Mean (SD)	*P* Value	Did Not Meet LLOS <72 (n = 7,521) Mean (SD)	Met LLOS ≥72 (n = 1,651) Mean (SD)	*P* Value
Age, y	79.4 (8.7)	78.6 (8.7)	80.2 (8.6)	∗<0.010	80.8 (8.7)	79.0 (8.6)	∗<0.010
Body mass index	28.3 (6.5)	28.3 (6.1)	28.3 (6.8)	0.72	28.3 (6.3)	28.1 (7.0)	0.24
Body surface area	1.88 (0.26)	1.9 (0.25)	1.86 (0.26)	∗<0.010	1.89 (0.25)	1.85 (0.27)	∗<0.010
Contrast volume (mL)	96.9 (52.3)	92.3 (45.1)	101.6 (58.6)	∗<0.010	94.7 (48.2)	106.6 (67.2)	∗<0.010
Five-meter walk test (seconds)	17.0 (22.9)	15.5 (22.6)	18.7 (23.1)	∗<0.010	16.3 (22.9)	20.5 (22.7)	∗<0.010
Glomerular filtration rate	66.8 (26.3)	69.5 (25.0)	64 (27.3)	∗<0.010	67.7 (25.8)	62.8 (28.1)	∗<0.010
Left ventricular ejection fraction (%)	58.5 (12.2)	59.2 (11.5)	57.8 (12.9)	∗<0.010	59.0 (11.9)	56.5 (13.5)	∗<0.010
KCCQ-12 score	53.6 (24.1)	56.9 (23.7)	50.1 (24.0)	∗<0.010	55.1 (24.0)	46.8 (23.4)	∗<0.010
Platelet count	206,982 (70,937)	206,367 (69,251)	207,634 (72,682)	0.39	206,886 (70,412)	207,418 (73,301)	0.79
Procedural duration (minutes)	90 (42.3)	83 (58.9)	94.3 (54.4)	0.23	85.3 (46.6)	105.6 (67)	∗<0.010
STS risk score	4.3 (3.5)	3.6 (2.9)	5.0 (3.9)	∗<0.010	4.0 (3.3)	5.6 (4.2)	∗<0.010

Categorical Predictors	N (%)	N	N		N	N	
Sex							
Male	5,241 (0.57)	2,876	2,365	∗<0.010	4,399	809	∗<0.010
Female	3,931 (0.43)	1,843	2,088	∗<0.010	3,122	842	∗<0.010
Procedure status				∗<0.010			∗<0.010
Not elective	270 (0.03)	54	216		160	110	
Elective	8,902 (0.97)	4,665	4,237		7,361	1,541	
Procedure location							
Cath lab	2,625 (0.29)	1,389	1,236	∗<0.010	2,210	415	∗<0.010
Hybrid cath lab/OR	6,547 (0.71)	3,330	3,217		5,311	1,236	
Access site							
Alternative access	315 (0.03)	79	236	∗<0.010	177	138	∗<0.010
Femoral	8,857 (0.97)	4,640	4,217		7,344	1,513	
Anesthesia type							
Moderate sedation	5,211 (0.57)	3,106	2,105	∗<0.010	4,520	691	∗<0.010
General anesthesia	3,961 (0.43)	1,613	2,348		3,001	960	
Access method							
Cut down	302 (0.03)	67	235	∗<0.010	165	137	∗;<0.010
Percutaneous	8,870 (0.97)	4,652	4,218		7,356	1,514	
Valve type							
Self-expanding	1,109 (0.12)	495	614	∗<0.010	853	256	∗<0.010
Balloon expanding	8,063 (0.88)	4,224	3,839		6,668	1,395	
Patient distance to TAVR center							
>50 miles	1,847 (0.2)	844	1,003	∗<0.010	1,485	362	∗<0.010
41-50 miles	430 (0.05)	218	212		353	77	
31-40 miles	751 (0.08)	371	380		623	128	
21-30 miles	873 (0.1)	438	435		708	165	
10-20 miles	1,737 (0.19)	926	811		1,435	302	
<10 miles	3,534 (0.39)	1,922	1,612		2,917	617	
Procedure day of week				0.33			0.40
Weekend	27 (0)	10	17		18	9	
Monday	1,944 (0.21)	1,012	932		1,633	311	
Tuesday	1,664 (0.18)	806	858		1,326	338	
Wednesday	2,886 (0.31)	1,496	1,390		2,361	525	
Thursday	1,821 (0.2)	951	870		1,501	320	
Friday	830 (0.09)	444	386		682	148	
Preop pacemaker	1,056 (0.12)	597	459		893	163	
Postop transfer to stepdown unit	5,504 (0.6)	3,699	1,805	∗<0.010	4,982	522	∗<0.010
Foley catheter (yes/no)	439 (0.05)	126	313	∗<0.010	286	153	∗<0.010
Pre-TAVR PFTs (yes/no)	1,324 (0.14)	497	827	∗<0.010	869	455	∗<0.010
Prior hospitalization in the last year	1,702 (0.19)	756	946	∗<0.010	1,291	411	∗<0.010
Discharge location (home)	8,689 (0.95)	4,667	4,022	∗<0.010	7,397	1,292	∗<0.010
Cath prior to TAVR	6,703 (0.73)	3,216	3,487	∗<0.010	5,394	1,309	∗<0.010
Long-term rhythm monitor	147 (0.02)	74	73	0.79	122	25	0.75
Mobile cardiac telemetry post-TAVR	87 (0.01)	38	49	0.09	66	21	0.11
Physical therapy post-TAVR	3,312 (0.36)	1,029	2,283	∗<0.010	2,215	1,097	∗<0.010
Major procedural complication	569 (0.06)	94	475	∗<0.010	225	344	∗<0.010
Minor procedural complication	525 (0.06)	126	399	∗<0.010	254	271	∗<0.010
Permanent pacemaker post- TAVR	553 (0.06)	75	478	∗<0.010	227	326	∗<0.010
PCI during TAVR admission	226 (0.02)	117	109	0.92	188	38	0.64
Atrial fibrillation/flutter	3,435 (0.37)	1,750	1,685	0.64	2,772	663	0.12
Diabetes	3,010 (0.33)	1,494	1,516	0.16	2,440	570	0.35
Prior stroke	861 (0.09)	391	470	0.25	672	189	0.33
Aortic regurgitation pre-TAVR				0.68			∗<0.010
Unrecorded	92 (0.01)	45	47		68	24	
Severe	386 (0.04)	206	180		307	79	
Moderate	1,325 (0.14)	688	637		1,075	250	
Mild	3,294 (0.36)	1,687	1,607		2,700	594	
Trace/Trivial	4,075 (0.44)	2,093	1,982		3,371	704	

KCCQ = Kansas City Cardiomyopathy Questionnaire; LLOS = long length of stay; OR = odds ratio; PCI = percutaneous coronary intervention; PFT = pulmonary functional test; SLOS = short length of stay; STS = Society of Thoracic Surgeons; TAVR = transcatheter aortic valve replacement.

∗ *P* < 0.05.

### Random forest model development and testing

**Figure 2:**
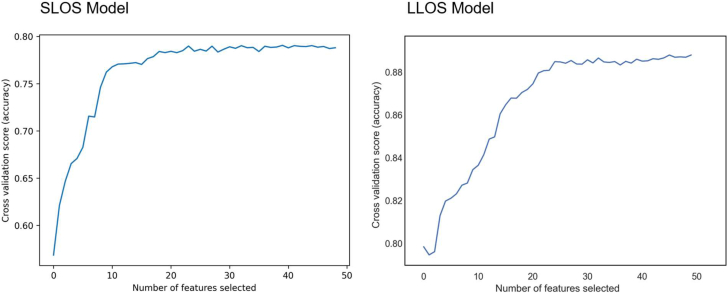
**Recursive Feature Elimination With Cross-Validation Graphs Demonstrating Relative Contribution of Each Variable in Model Prediction** Recursive feature elimination with cross-validation (RFECV) demonstrating the relative contribution of each variable to model performance. This technique identifies the most influential predictors by iteratively removing less important features.

**Figure 3:**
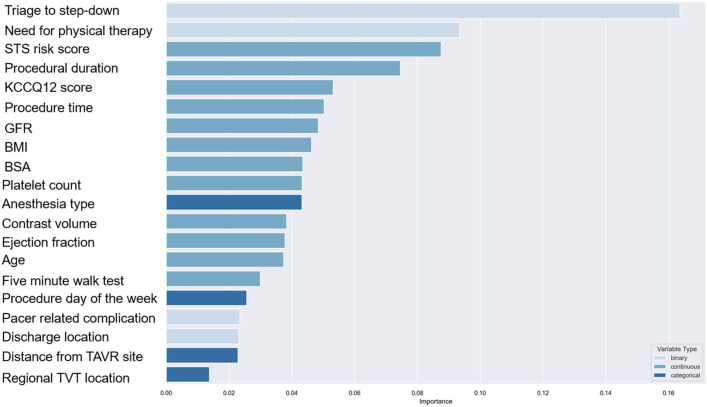
**Relative Importance Plot for Major Predictors of Short Length of Stay (<36 Hours)** Relative importance plot highlighting key variables associated with short length of stay (SLOS), defined as <36 hours. The top predictors were ranked by their impact on the model's predictive accuracy BMI = body mass index; BSA = body surface area; GFR = glomerular filtration rate; KCCQ = Kansas City Cardiomyopathy Questionnaire; STS = Society of Thoracic Surgeons; TAVR = transcatheter aortic valve replacement; TVT = Transcatheter Valve Therapy.

Initial model development identified 44 total significant predictors for SLOS and 45 predictors for LLOS (Supplemental Tables 1 and 2). RFECV curves were used to develop more parsimonious models, with predictive power plateauing at 20 predictors for SLOS and 22 predictors for LLOS (Figure 2). For SLOS, the most important predictors were triage to intensive care unit (ICU) vs step-down unit post-TAVR, followed by procedural start time, STS risk score, procedure duration, use of physical therapy post-TAVR, KCCQ12, body mass index (BMI), platelet count, glomerular filtration rate, body surface area (BSA), contrast usage, left ventricular ejection fraction, patient age, and anesthesia type used (Figure 3). When run using the test cohort, sensitivity for the SLOS model was 0.81 with a specificity of 0.70 and an AUC of 0.82. In the multivariate logistic model using the same variables to predict SLOS (Supplemental Table 1), the AUC was 0.65.

**Figure 4:**
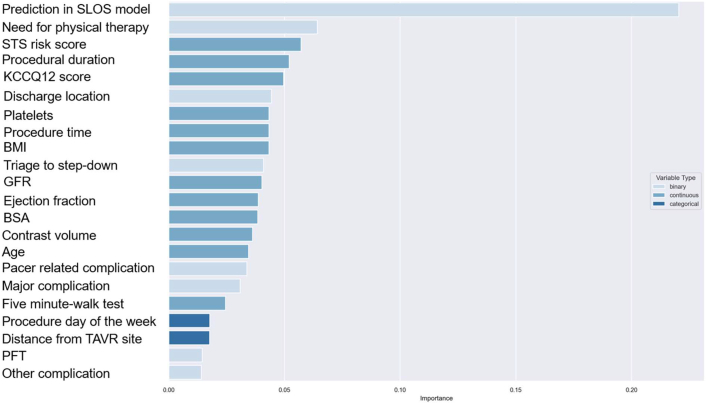
**Relative Importance Plot for Major Predictors of Long Length of Stay (>72 Hours)** Relative importance plot for variables most strongly associated with long length of stay (LLOS), defined as >72 hours. Predictors are ranked by their influence on the model's output PFT = pulmonary functional test; SLOS = short length of stay; other abbreviations as in Figure 3.

For LLOS, the most powerful predictors, after the SLOS model prediction, were use of physical therapy followed by STS risk score, procedure duration, KCCQ12, discharge location, platelet count, and BMI (Figure 4). Sensitivity for the LLOS model was 0.45 with a specificity of 0.94 and an AUC of 0.85. In the multivariate logistic model using these variables to predict LLOS (Supplemental Table 2), the AUC was 0.65.

**Figure 5:**
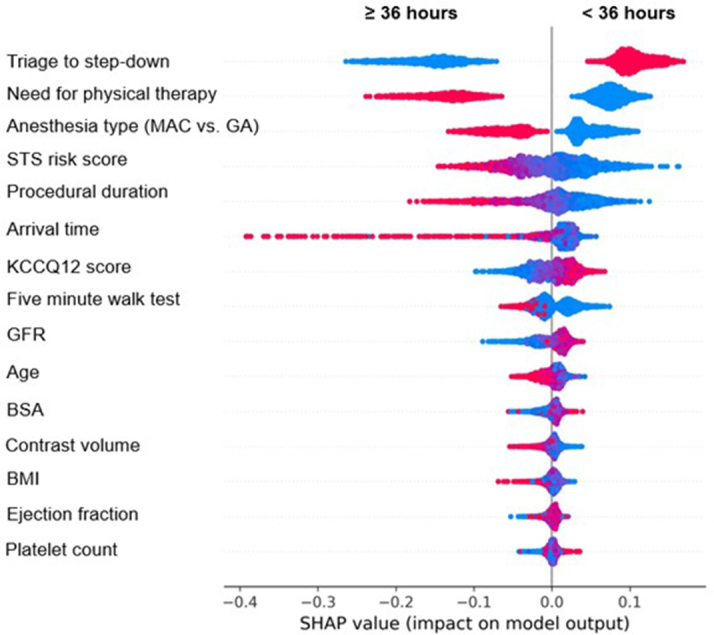
SHAP Values for Predictors of SLOS For binary variables, red = yes and blue = no. For continuous variables, red values = higher values. SHAP (SHapley Additive exPlanations) summary plot for predictors of SLOS. Red indicates presence of a binary feature or higher value of a continuous feature; blue indicates absence or lower value, respectively. GA = general anesthesia; MAC = monitored anesthesia care; other abbreviations as in Figure 3 and 4.

**Figure 6:**
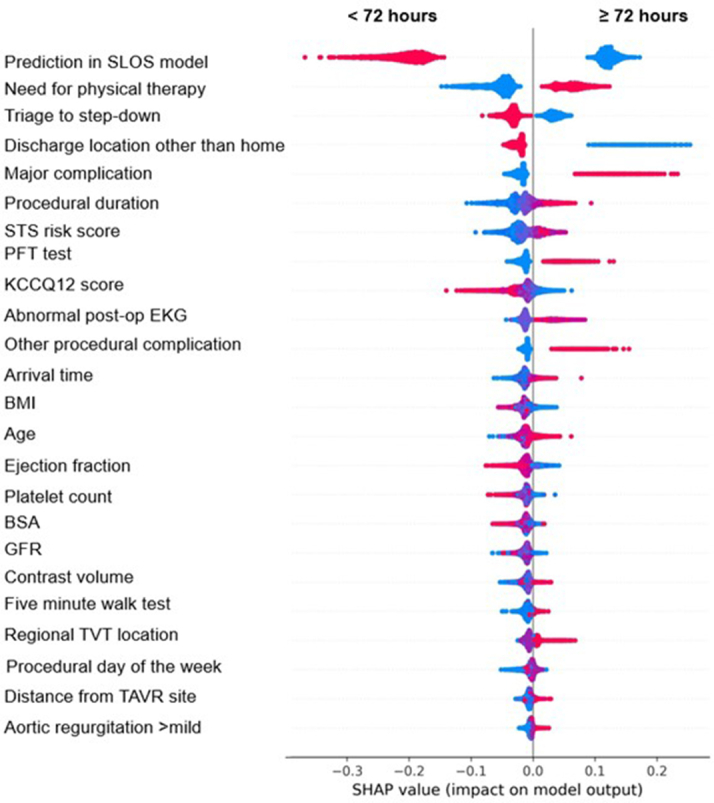
**SHAP Values for Predictors of LLOS** For binary variables, red = yes and blue = no. For continuous variables, red values = higher values. SHAP summary plot for predictors of LLOS. As with SLOS, red and blue indicate the presence or absence (or relative value) of binary and continuous features, respectively. EKG = electrocardiogram; LLOS = long length of stay; SHAP = SHapley Additive exPlanations; other abbreviations as in Figures 3 and 4.

Corresponding directionality of each predictor in the model is represented by SHapley Additive exPlanations values for SLOS (Figure 5) and LLOS (Figure 6). For SLOS, triage to step-down as opposed to ICU post-TAVR, no need for physical therapy post-TAVR, and no use of general anesthesia were all directionally associated with meeting SLOS cutoffs. Higher STS risk score, longer procedural duration, lower KCCQ-12 score, need for permanent pacer post-TAVR, lower glomerular filtration rate, lower BSA, lower ejection fraction, lower platelet count, a discharge location to any location besides home, slower performance on 5-m walk test, longer distance from home address to TAVR hospital, increasing age, procedures performed later in the week, procedures performed in an operating room (hybrid or standard) vs catheterization (hybrid or standard) laboratory settings were all associated with not meeting SLOS. Procedural start time earlier in the day was associated with not meeting SLOS cutoffs.

**Figure 7:**
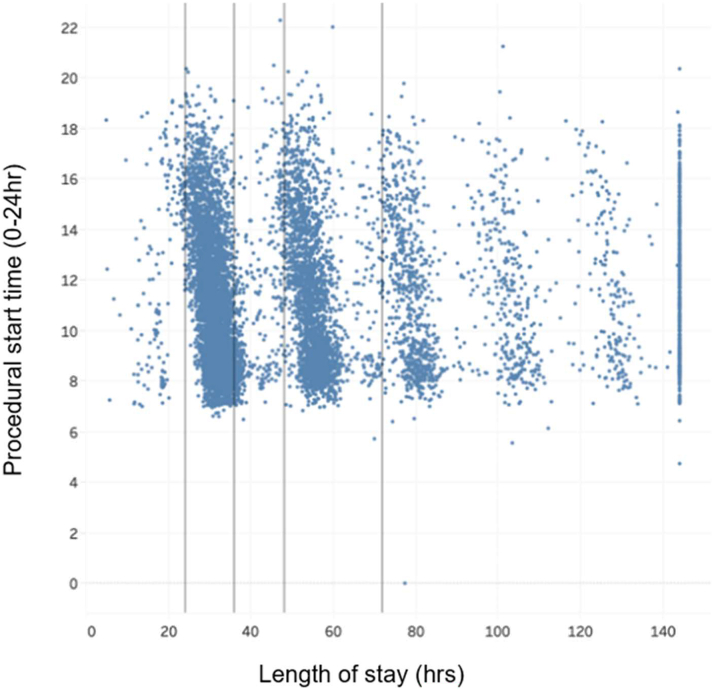
**Procedural Start Time Vs Length of Stay** Scatterplot of procedural start time vs length of stay. The plot explores potential associations between the timing of procedures and hospital LOS across the BIOME data set LOS = length of stay.

For LLOS, prediction for SLOS in the prior model, need for physical therapy post-TAVR, discharge location to any location besides home, triage directly to ICU post-TAVR, need for permanent pacemaker post-TAVR and major procedural complications were most directionally associated with LLOS. Higher STS risk score, longer procedural duration, pre-TAVR pulmonary functional test, worse performance on KCCQ-12, lower ejection fraction, minor procedural complications, lower platelet count, later procedural time, lower BMI and BSA, increasing age, higher volumes of contrast administered, and longer distance from home to TAVR hospital were also associated with LLOS. Procedures performed earlier in the week (Monday defined as first day of the week, Sunday last) and those patients who performed better on the 5-m walk test were less likely to have LLOS.

Additional sensitivity analysis was performed on procedural start time given the unexpected directionality of the association with SLOS (ie, earlier procedural start time was associated with not meeting SLOS cutoff). Figure 6 demonstrates that across the entire data set, earlier procedural start time tends to associate with longer LOS, with clustering effects between hours 24 to 36, and between 50 to 72 hour and relatively fewer patients discharged before 24 hours or between 36 to 50 hours. Given that patients who undergo outpatient TAVR later in the day may not be admitted until later compared to those patients undergoing earlier morning TAVR, additional analysis was performed examining the relative contributions of pre- and post-TAVR LOS times to those undergoing morning vs afternoon TAVR procedures. Table 2 demonstrates the relative contributions to LOS among before and afternoon TAVR across LOS cohorts. Average start time for the before noon group was 9:00 AM and for the afternoon group was 2:00 PM among SLOS and in the overall cohort. Among the SLOS group, the average LOS preprocedure was shorter for TAVR procedures performed before noon (3 hours) compared to the afternoon group (5 hours) but with a corresponding increase in postprocedural LOS in the SLOS cohort (28 vs 24 hours) leading to a net overall increased LOS among the SLOS cohort (31 vs 29 hours).

**Table 2:** Breakdown of Length of Stay Stratified by SLOS Status and Procedural Start Time

	Procedure Start Time Before 12:00 h	Procedure Start Time After 12:00 h
Met SLOS cutoff		
N	3,239	1,586
Avg. procedure start time + postprocedure LOS h	38	39
Avg. procedure start time	09:00	14:00
Avg. postprocedure LOS	28	24
Avg. LOS preprocedure h	3	5
Avg. LOS h	31	29
Did not meet SLOS cutoff		
N	2,897	1,638
Avg. procedure start time + postprocedure LOS h	88	93
Avg. procedure start time	09:00	14:00
Avg. postprocedure LOS	79	78
Avg. LOS preprocedure h	5	6
Avg. LOS h	83	84
All cases		
N	6,136	3,224
Avg. procedure start time + postprocedure LOS h	61	66
Avg. procedure start time	09:00	14:00
Avg. postprocedure LOS	52	52
Avg. LOS preprocedure h	4	5
Avg. LOS h	56	57

Avg = average; LOS = length of stay; other abbreviation as in Table 1.

Before or after 12:00 hours rounded to the nearest hour.

## Discussion

Among 9,172 patients in the Biome registry who underwent outpatient TAVR between 2017 and 2021, a novel ML algorithm outperformed a standard multivariable model in predicting both SLOS and LLOS and identified several novel predictors of LOS.

Twenty variables were important in predicting SLOS, while 22 variables were included in the LLOS model. For predicting SLOS, over 50% of the predictive power of the model was captured with 6 variables. These included triage to step-down as opposed to ICU post-TAVR, no need for physical therapy consult post-TAVR, lower STS risk score, shorter procedural duration, higher KCCQ-12 scores, and later procedural start time. The LLOS provided less sensitivity than the SLOS model (0.45 vs 0.81), but a higher level of specificity (0.94 vs 0.70).

Similarly, in predicting LLOS, over 50% of the predictive power of the model was captured with 6 variables. SLOS prediction from the first model, requiring physical therapy post-TAVR, higher STS risk score, longer procedural duration, lower KCCQ-12 scores, and discharge to a location other than home were the most important variables in the LLOS model.

We also found a clear clustering effect whereby LOS was centered around 24 to 36 hours and from 48 to 72 hours with significantly fewer discharges after 72 hours. Procedural start time earlier in the day was also associated with not meeting SLOS cutoff. Among those patients who met SLOS cutoff and had earlier procedural start times, preprocedural LOS was slightly shorter, but postprocedural LOS was longer compared to those patients who had later procedural start times. There were no significant differences between preprocedural and postprocedural LOS in patients who did not meet SLOS, indicating that the association between procedural start time and meeting SLOS cutoffs is likely not driven by differences in preprocedural and postprocedural LOS. These differences are possibly due to unmeasured confounding and inherent differences between those patients who undergo TAVR earlier in the day vs later.

LOS following TAVR has broad implications for hospitals and health care systems worldwide as TAVR has become the predominant form of aortic valve replacement in the United States and demand is expected to grow with an aging population.^2^ Identifying both patient and procedural characteristics that may influence LOS have been of keen interest in the literature since the FDA approval of TAVR in 2011. In regard to patient factors, advanced age, female sex, chronic kidney disease, heart failure, and atrial fibrillation have all been linked to prolonged LOS.^6^, ^7^, ^8^, ^9^, ^10^ Various markers of frailty including walk tests, grip strength, serum albumin, activity of daily living assessments, skeletal muscle indices, and preoperative mental acuity tests have been found to influence LOS among others.^11^, ^12^, ^13^ Reduced pre-TAVR left ventricular function and low mean aortic valve gradients have been implicated in delayed discharge.^8^^,^^14^ Environmental and socioeconomic factors may also influence LOS, with studies identifying non-Caucasian race and lower income with prolonged LOS following TAVR.^8^ The ability to discharge a patient to home following TAVR has been associated with shorter LOS.^15^ Procedural and hospital-level factors have also been identified as important predictors of LOS following TAVR. Nonfemoral access or need for surgical cut-down have all been associated with prolonged LOS.^9^ TAVR resulting in need for permanent pacemaker or resulting in other complications, the use of general anesthesia, and self-expanding valves have all been associated with prolonged LOS.^16^ So called “minimalist” approaches to TAVR aimed at limiting use of general anesthesia, performing procedures in catheterization laboratories as opposed to operating rooms, and avoiding Foley catheter placement, have all been associated with shorter LOS without increases in complications or mortality.^7^^,^^17^

Our findings are consistent with previous research on the LOS post-TAVR and add to the literature in several ways. First, the use of a ML algorithm for predicting LOS post-TAVR has not been reported. ML algorithms have been used to predict total hospitalization costs during TAVR, but LOS was the primary predictor of increased cost.^18^ ML algorithms have several potential advantages over standard multivariable regression approaches. A supervised random forest model can estimate the relative importance of various parameters in predicting an outcome and may have higher predictive power than standard multivariable approaches, as was seen in our analysis. Additionally, our study provides information as to the relative importance of specific variables in the prediction of LOS as well as their direction. Triage to step-down bed as opposed to ICU, lack of need for physical therapy post-TAVR, lower STS risk scores, shorter procedural duration, and higher KCCQ-12 scores were ranked the most important predictors for achieving a SLOS. Results were similar for predicting LLOS; however, sensitivity was reduced with high specificity likely due to reduced numbers of cases in the LLOS category. Novel predictors of LOS in this study include need for physical therapy post-TAVR, procedure duration, procedure start time, procedure day of the week, and distance of the patient’s home address to TAVR hospital.

Strengths of this study include its large size, multicenter involvement, predictors included, and use of a random forest model for prediction.

### Study limitations

Results of this study should be viewed in the context of several limitations. In multivariable analyses of retrospective data, unmeasured confounding may influence the effect of predictors on the outcome. For example, the finding of physical therapy consults being linked to longer LOS may reflect the increased frailty of these patients, which is not fully captured by our data. Additionally, the triage decision to step-down or ICU may be confounded by patient and procedural factors that may not be accurately captured in our data. Our study also relies on internal validation against the test data set and is not externally validated. Finally, as a requirement for its use, the random forest model requires imputation of missing data, although this was a small amount of the overall data set.

## Conclusions

ML analysis of over 9,000 patients in the multicenter Biome data set provided insights into the relative importance of various predictors in post-TAVR LOS. Several novel predictors were found to influence LOS, including need for physical therapy, procedure duration, procedure start time, and procedure day of the week. Triage to stepdown or ICU post-TAVR was the most important predictor for SLOS. Future studies may be focused on interventions to improve LOS following TAVR in patients at higher risk of prolonged LOS.

Perspectives **COMPETENCY IN: SYSTEMS-BASED PRACTICE:** TAVR is a rapidly expanding procedure with increasing adoption rates. According to the latest Transcatheter Valve Therapy National Cardiovascular Data Registry data, 72,991 TAVR procedures were performed last year, highlighting its growing acceptance. With over 700 centers in the United States now performing TAVR, the procedure imposes a significant economic burden. To address this, we developed a ML algorithm to enhance cardiovascular care by predicting the LOS post-TAVR. The algorithm's predictive power surpassed that of standard multivariable models by revealing procedural duration, postprocedure physical therapy, and procedure day of the week as important predictors. This ML tool shows potential opportunity for cost-effective measures for large volume procedures reducing cost through better resource management.

**TRANSLATIONAL OUTLOOK:** ML algorithms have an important role in identifying novel predictors of short and prolonged lengths of stay following TAVR, facilitating targeted quality improvement programs. Further validation would be required before widespread clinical adoption.

## Funding support and author disclosures

This analysis was funded by an institutional grant between the University of California-San Francisco and Biome Analytics. The authors have reported that they have no relationships relevant to the contents of this paper to disclose.
