# Supplementary material for: Predictors of Length-of-Stay Among Transcatheter Aortic Valve Replacement Patients Using a Supervised Machine Learning Algorithm
Source: JACC Adv. 2025 Jun 24;4(8):101902. doi: 10.1016/j.jacadv.2025.101902 (PMC12256322; doi:10.1016/j.jacadv.2025.101902)
Supplement: Supplemental_Material [file mmc1.docx]

**Supplemental Table 1. Logistic regression model for short length of stay (SLOS)**

| Predictor | OR | CL lower bound | CL upper bound | P>\|z\| | Gini rank |
| --- | --- | --- | --- | --- | --- |
| Direct to step down unit | 4.10 | 3.62 | 4.63 | <0.01 | 1 |
| Physical therapy consult | 0.30 | 0.27 | 0.35 | <0.01 | 2 |
| STS risk score | 0.94 | 0.91 | 0.96 | <0.01 | 3 |
| Procedural duration | 1.00 | 1.00 | 1.00 | <0.01 | 4 |
| KCCQ12 score | 1.01 | 1.00 | 1.01 | <0.01 | 5 |
| Procedure start time | 1.02 | 1.00 | 1.04 | 0.40 | 6 |
| GFR | 1.00 | 1.00 | 1.01 | 0.01 | 7 |
| BSA | 1.29 | 0.99 | 1.68 | 0.06 | 8 |
| BMI | 0.99 | 0.97 | 1.00 | 0.02 | 9 |
| Platelets | 1.00 | 1.00 | 1.00 | 0.42 | 10 |
| Anesthesia type | 0.48 | 0.42 | 0.55 | <0.01 | 11 |
| Contrast volume | 1.00 | 1.00 | 1.00 | 0.01 | 12 |
| Ejection fraction | 1.00 | 1.00 | 1.01 | 0.86 | 13 |
| Age | 0.99 | 0.98 | 0.99 | <0.01 | 14 |
| Five minute walk test | 1.00 | 0.99 | 1.00 | <0.01 | 15 |
| Procedure day of the week | 0.95 | 0.90 | 0.99 | 0.02 | 16 |
| PPM needed | 0.20 | 0.14 | 0.27 | <0.01 | 17 |
| Discharge location | 4.12 | 2.94 | 5.77 | <0.01 | 18 |
| Home distance from TAVR center | 0.91 | 0.88 | 0.93 | <0.01 | 19 |
| Regional TVT location | 0.76 | 0.65 | 0.89 | <0.01 | 20 |

**Supplemental Table 2. Logistic regression model for long length of stay (LLOS)**

| Predictor | OR | CL lower bound | CL upper bound | P>\|z\| | Gini rank |
| --- | --- | --- | --- | --- | --- |
| SLOS prediction | 0.08 | 0.06 | 0.10 | <0.01 | 1 |
| Physical therapy consult | 1.75 | 1.47 | 2.09 | <0.01 | 2 |
| STS risk score | 1.00 | 0.98 | 1.02 | 0.90 | 3 |
| Procedure duration | 1.00 | 1.00 | 1.00 | 0.10 | 4 |
| KCCQ12 score | 0.99 | 0.99 | 1.00 | <0.01 | 5 |
| Discharge location | 0.19 | 0.14 | 0.25 | <0.01 | 6 |
| Platelets | 1.00 | 1.00 | 1.00 | 0.45 | 7 |
| Procedure time | 1.01 | 0.98 | 1.04 | 0.52 | 8 |
| BMI | 0.99 | 0.98 | 1.01 | 0.29 | 9 |
| Direct to step down unit | 0.86 | 0.72 | 1.04 | 0.12 | 10 |
| GFR | 1.00 | 1.00 | 1.00 | 0.39 | 11 |
| Ejection fraction | 0.99 | 0.98 | 1.00 | <0.01 | 12 |
| BSA | 0.97 | 0.67 | 1.40 | 0.86 | 13 |
| Contrast volume | 1.00 | 1.00 | 1.00 | 0.93 | 14 |
| Age | 1.01 | 1.00 | 1.02 | 0.01 | 15 |
| PPM needed | 4.95 | 3.81 | 6.43 | <0.01 | 16 |
| Major complication | 3.65 | 2.72 | 4.90 | <0.01 | 17 |
| Five minute walk test | 1.00 | 1.00 | 1.00 | 0.87 | 18 |
| Procedure day of the week | 1.10 | 1.03 | 1.17 | 0.01 | 19 |
| Home distance from TAVR center | 1.03 | 0.98 | 1.07 | 0.24 | 20 |
| Pulmonary function test results | 2.29 | 1.86 | 2.81 | <0.01 | 21 |
| Other procedural complication | 2.77 | 2.04 | 3.77 | <0.01 | 22 |
